# Supplementary figures and images for: Calcification detection on upper extremity arteries: a comparison of ultrasonic and X-ray methods
Source: PeerJ. 2023 Aug 23;11:e15855. doi: 10.7717/peerj.15855 (PMC10460149; doi:10.7717/peerj.15855)

## Imaging data

### Case 1

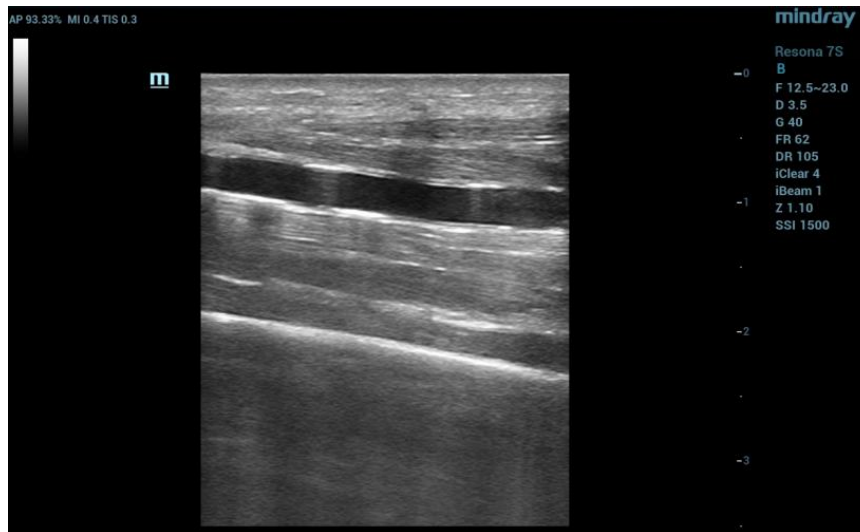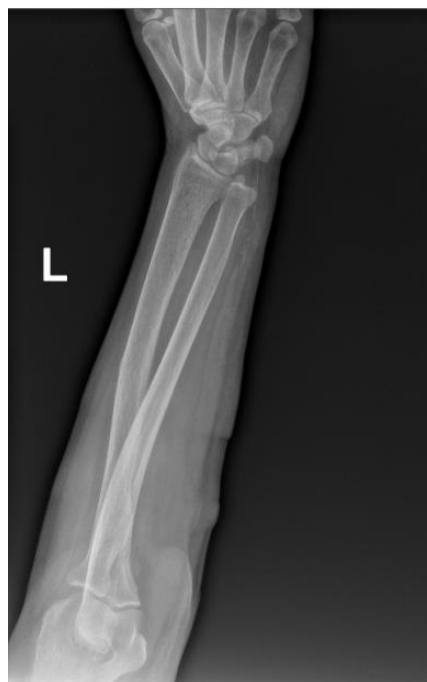

## Case 2

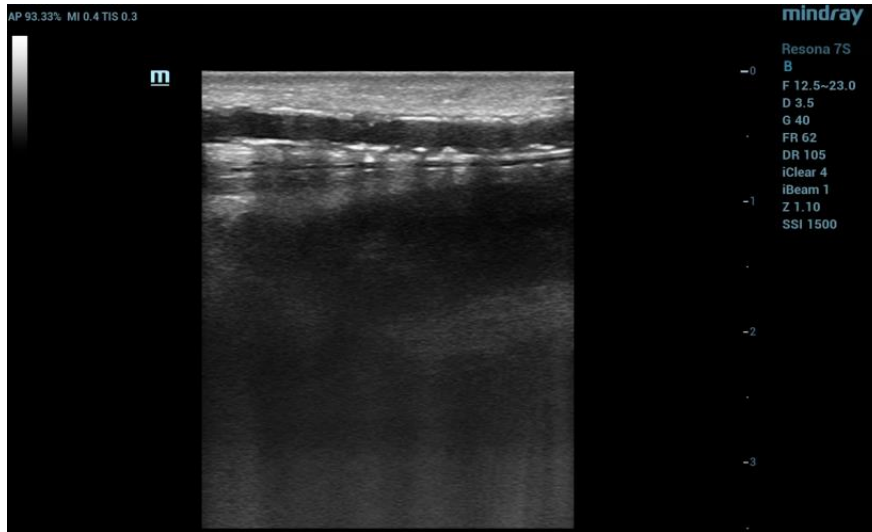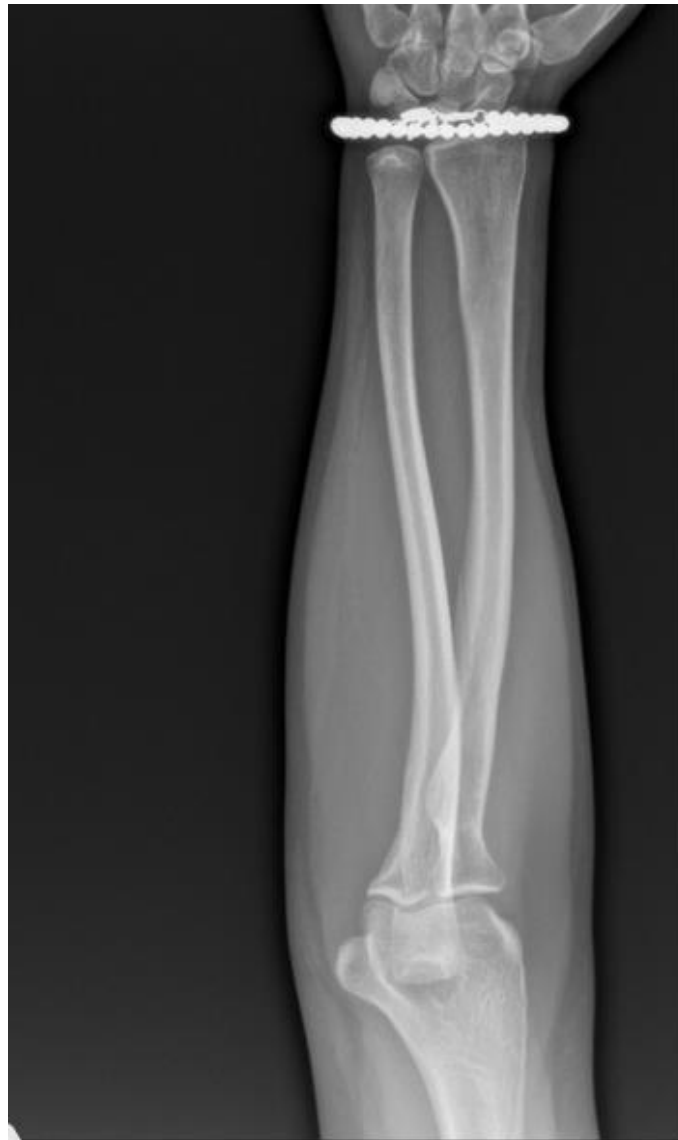

## Case 3

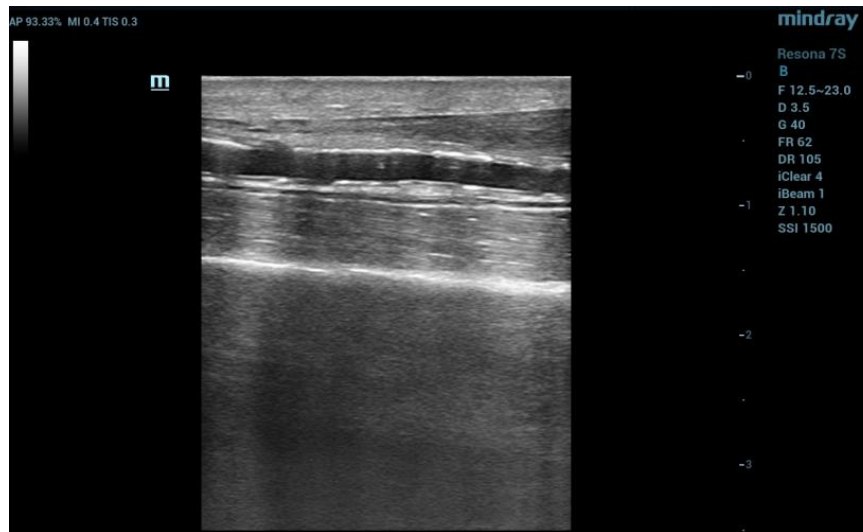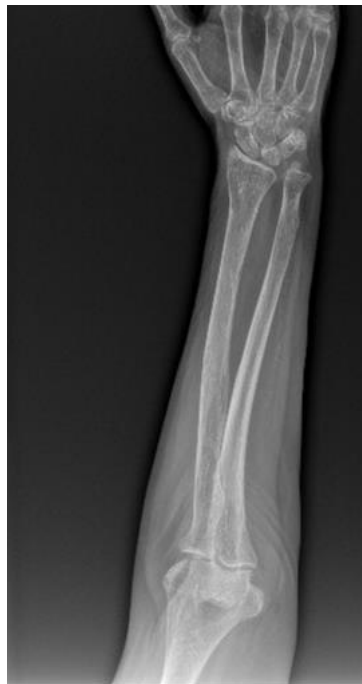

## Case 4

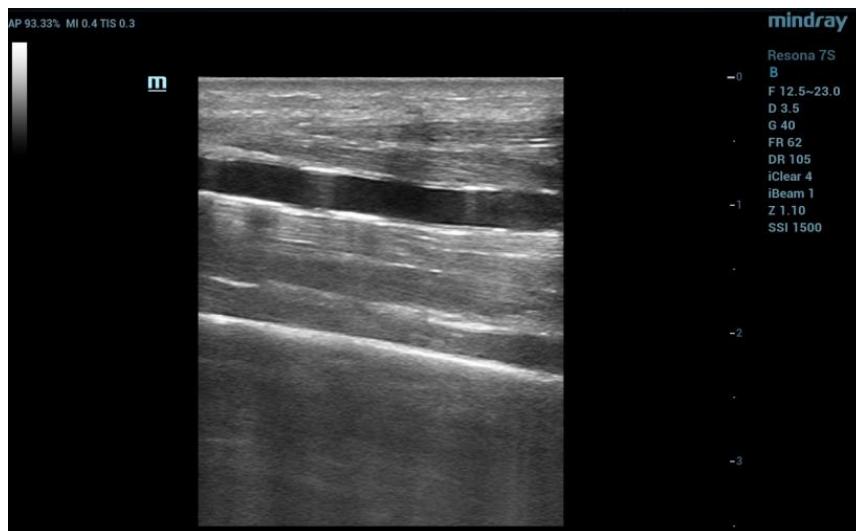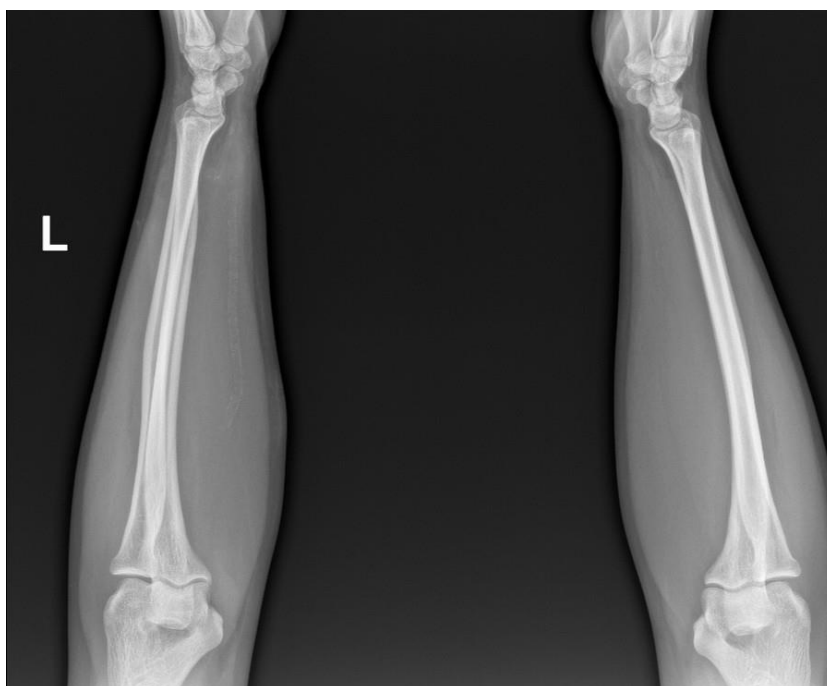

Supplement: Supplemental Information 2 [file peerj-11-15855-s002.pdf]
