# Supplementary material for: Calcification detection on upper extremity arteries: a comparison of ultrasonic and X-ray methods
Source: PeerJ. 2023 Aug 23;11:e15855. doi: 10.7717/peerj.15855 (PMC10460149; doi:10.7717/peerj.15855)
Supplement: Supplemental Information 4 [file peerj-11-15855-s004.pdf]

Protocol

1. Patients were enrolled in the study.
2. Research staff collected the information about demographic information and clinical characteristics, including age, gender, body mass index, hemodialysis duration, AVF duration, primary renal disease, dialysis clearance, and history of diabetes, hypertension, cardiovascular disease, and cerebral stroke.
3. The research nurses collect blood sample for laboratory.
4. Ultrasound examination:
   (1). Doppler diagnostic ultrasound system (Resona7s, Shenzhen, China) with linear array transducers (L20-5u 13.5MHz
   (2). Participants were set in the sitting position, and B-mode ultrasound was used to observe the anatomy and determine calcification lesions' presence in transverse and longitudinal sections.
   (3). Calcification lesions criteria: a localized echo structure of a hyperechogenic spot with posterior shadowing lining the vessel walls, either in an irregular pattern, a punctate pattern, or a linear pattern.
5. X-ray examination:
   (1) Radiograph machine (MULTI X fusion Max, Siemens, German).
   (2). On the posterior-anterior and lateral positions, plain X-ray of the upper extremities were obtained
   (3). Calcification lesions criteria: any lesion lining the vessel walls with **high-density shadow,** either in an irregular pattern, a punctate pattern, or a linear pattern.
6. Data analysis on the detection consistency in calcification between ultrasound and X-ray.
